# Comparison of Dexmedetomidine with Midazolam as an adjuvant with Propofol for insertion of ProSeal laryngeal mask airway in Children

**DOI:** 10.5152/TJAR.2023.21428

**Published:** 2023-04-01

**Authors:** Pooja Gunwal, Sapna Bathla, Anju Kumari, Jeetendra Kumar Bajaj

**Affiliations:** 1 Department of Anaesthesiology and Intensive Care, VMMC & Safdarjung Hospital, New Delhi

**Keywords:** Dexmedetomidine, midazolam, paediatric, ProSeal laryngeal mask airway

## Abstract

**Objective:**

Propofol is required in higher doses for smooth insertion of the ProSeal laryngeal mask airway. The ideal adjuvant drug so as to minimise induction doses of propofol is still not known. Dexmedetomidine and midazolam are equally effective for premedication in children. We have designed this study to compare dexmedetomidine and midazolam as adjuvants with propofol for insertion characteristics of ProSeal laryngeal mask airway.

**Methods:**

A total of 130 paediatric patients undergoing elective surgery were randomly allocated into 2 groups of 65 each. One group was induced using propofol, fentanyl and midazolam, whereas the other group received propofol, fentanyl and dexmedetomidine. Subsequently, insertion characteristics of ProSeal laryngeal mask airway were documented in terms of number of attempts and by using modified Muzi score. Post-operative sedation was recorded by Ramsay Sedation Scale and pain was assessed by using Wong–Baker Faces pain scale.

**Results:**

Out of 130 patients, ProSeal laryngeal mask airway was inserted in a second attempt in only 5 patients of midazolam group. Time taken for insertion was significantly higher among the midazolam group (21 seconds) than the dexmedetomidine group (19 seconds). A total of 93.8% of patients administered dexmedetomidine had excellent Muzi scores in comparison to midazolam group where only 13.8% patients had excellent Muzi scores (*P* < .001).

**Conclusion:**

Dexmedetomidine in a dose of 1 µg kg^–1^ as compared to midazolam (20 µg kg^–1^) produces better insertion characteristics for ProSeal laryngeal mask airway when used as adjuvant with propofol in terms of jaw opening, ease of insertion, coughing, gagging, patient movement, and laryngospasm.

**Main Points**

- A variety of pharmacological agents have been tried to overcome side effects associated with ProSeal laryngeal mask airway (PLMA) insertion, for example fentanyl, intravenous lignocaine, ketamine and clonidine but none have been found to be ideal.
- We hypothesised that dexmedetomidine was better than midazolam as an adjuvant with propofol for insertion of PLMA and hemodynamic stability.
- Jaw opening, ease of insertion and body movements score were significantly higher in dexmedetomidine group.
- Dexmedetomidine (at dose 1 μg kg^-1^) produces better insertion characteristics.
- It also provides better haemodynamic stability intra-operatively and better sedation and smooth emergence post-operatively as compared to midazolam.

## Introduction

The ProSeal laryngeal mask airway (PLMA) has gained widespread popularity for airway management during surgery. Endotracheal intubation, the gold standard for securing airway, is an invasive measure and associated with greater hemodynamic alteration, whereas PLMA serves as a bridge between the facemask and endotracheal tube and is good alternative for short duration procedures as it is less invasive and less stimulating, hence preventing stress response. ProSeal laryngeal mask airway is a modification of classic laryngeal mask airway (cLMA) that incorporates a drain tube ending at the tip of mask so that there are less chances of aspiration. Use of muscle relaxant is not essential for PLMA insertion, although a certain degree of jaw relaxation and depth of anaesthesia is required.

Propofol is a useful induction agent for PLMA insertion because of its properties of producing good jaw relaxation and suppression of the airway reflexes.^1^ When used alone as an intravenous induction agent, the dose of propofol often exceeds 2.5 mg kg^-1^, and easy insertion was seen only in about 62% of patients.^2^ Undesired side effects such as drop in blood pressure, coughing, laryngospasm and body movement may occur.^2^

A variety of pharmacological agents have been tried to overcome these side effects and lead to easy PLMA insertion, for example fentanyl,^3^ intravenous lignocaine,^4^ ketamine^5^ and clonidine^6,7^ but none have been found to be ideal.

Midazolam and propofol both are known to act synergistically on gamma amino butyric acid-A (GABA-A) receptors and also reduce dose requirement.^8^ Midazolam is a short acting benzodiazepine; causes anterograde amnesia and thus reduces the risk of awareness. Bhaskar et al^8^ in his studies reported the effect of midazolam premedication on the dose of propofol for laryngeal mask airway insertion in children. They found that midazolam is an effective premedicant where it is synergistic with propofol and reduces its effective dose requirement for PLMA insertion in children.

Dexmedetomidine is a highly selective α-2 adrenergic agonist. Its action at α-2 adrenoreceptors on locus ceruleus is responsible for its sedative properties. The sympatholytic effect and slight sedation is also due to activation of post-synaptic receptors in central nervous system and analgesic effect comes from binding to α-2 receptors present in spinal cord. Intravenous and intranasal^9^ dexmedetomidine is being used as a premedication with benefits of sedation, analgesic, anxiolysis and hemodynamic stability.^9^ Its effects are dose dependent and do not cause respiratory depression at clinically effective dosages. It is also shown to depress airway and circulatory response.

So both dexmedetomidine and midazolam can be used as adjuvants to propofol which decreases the incidence of adverse responses to insertion of PLMA. We considered the use of either of these drugs given before propofol may obtund airway reflexes sufficiently to allow satisfactory insertion of PLMA. We have compared the use of dexmedetomidine and midazolam as an adjuvant with propofol for insertion characteristics of PLMA. There is paucity of literature comparing dexmedetomidine–propofol and midazolam–propofol for PLMA insertion characteristics in paediatric age group. It was hypothesised that dexmedetomidine is better than midazolam as an adjuvant with propofol for insertion of PLMA and hemodynamic stability. The primary objective was to compare ease of insertion (by using first attempt success rate and time taken for insertion) of PLMA in spontaneously breathing children using dexmedetomidine and midazolam as co-induction agent with propofol. The secondary objective was to compare the hemodynamics and oxygenation at insertion, recovery characteristics and pain.

## Materials and Methods

**Figure 1.**
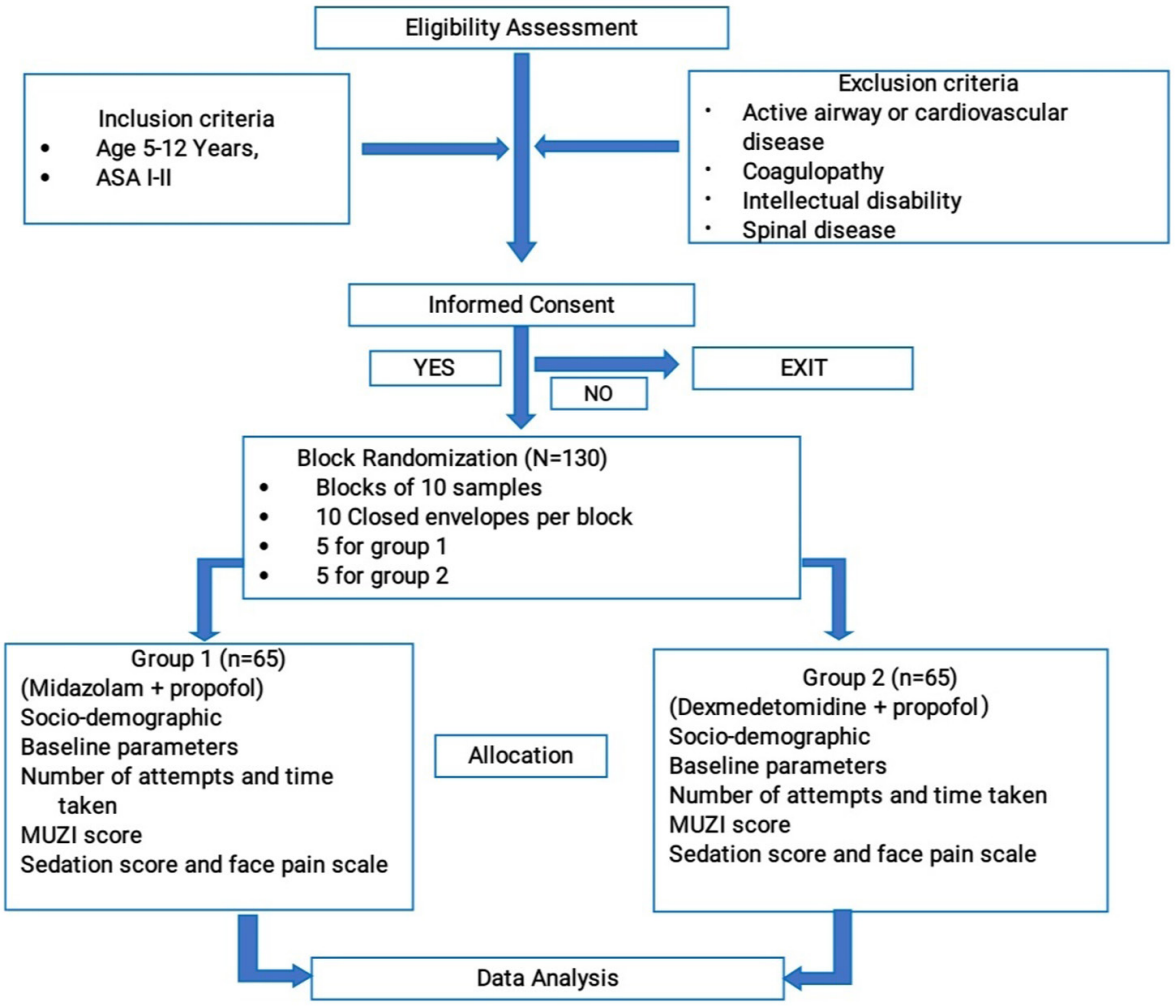
CONSORT flowchart.

After obtaining ethical clearance from the institute, ethics committee and informed consent from patients, 130 paediatric patients (5-12 years) with American Society of Anaesthesiology (ASA) grade I and II of either gender were allocated randomly into 2 groups of 65. Pre-anaesthetic evaluation was done, and investigations were done as per departmental protocol. Patients with any known drug allergies, history of active airway disease or cardiovascular disorder, congenital or acquired coagulopathy, developmental delay (not able to comprehend or communicate verbally) and anticipated difficult airway were excluded. Patients were randomly allocated into 2 groups using block randomisation with sealed envelope system (Figure 1). Block randomisation was done with sealed envelope system. In this, 10 randomly generated treatment allocations were prepared with sealed opaque envelope assigning 1 and 2 in 5 envelope each where 1 represented group 1 receiving midazolam with propofol and 2 represented group 2 receiving dexmedetomidine with propofol. After taking informed consent from parents, an envelope was opened and the patient was then allocated the group. Envelope was opened by the person not involved in the study.

All children were pre-medicated with oral triclofos (50mg kg^-1^) 2 hours prior to procedure. Venous cannulations were done once the child was sedated after sleep was induced and within 1 hour of receiving triclofos. Group 1 received intravenous midazolam 20 µg kg^–1^ diluted in 10 mL normal saline infused over 5 minutes and group 2 received intravenous dexmedetomidine 1 µg kg^–1^ diluted in 10 mL normal saline infused over 5 minutes. Thirty seconds later fentanyl 1 µg kg^–1^ was given to both the groups. After 30 seconds propofol 2.5 mg kg^–1^ was given for induction. After loss of verbal contact and loss of eyelash reflexes, the children were ventilated with 100% oxygen for 60 seconds. Then, after 60 seconds, PLMA insertion was attempted and grading of insertion characteristics was done. Time taken for insertion and number of attempts were noted along with hemodynamic parameters. Documentation was being done at following time intervals; T0—baseline parameters, T1—immediately after study drug, T2—immediately after propofol induction, T3—immediately after PLMA insertion, T4—after 1 minute of PLMA insertion, T5—after 3 minutes of PLMA insertion, T6—after 5 minutes of PLMA insertion, T7—after 10 minutes of PLMA insertion. The insertion characteristics were noted using modified Muzi score which includes various parameters of introduction of PLMA (jaw opening, ease of insertion) and patient response (coughing, gagging, patient movement, and laryngospasm) (Table 1).^10,11^ Effective ventilation was confirmed by chest movements and square wave capnography. After 2 attempts, if PLMA insertion was not successful, the airway device was changed to endotracheal tube, and the case was removed from the study. Anaesthesia was maintained with 1%-3% sevoflurane with nitrous oxide and oxygen. At the end of surgery, patients were monitored for complete recovery. Post-operative sedation was recorded using Ramsay Sedation Scale and pain was assessed by using Wong–Baker FACES pain scale.

Statistical analysis was done using Statistical Package for Social Sciences (SPSSv26.0). Sample size was calculated with 80% power of study and 5% level of significance based on observation of first attempt success rate for PLMA insertion in propofol with midazolam and propofol with dexmedetomidine was 84% and 98%, respectively.^12^ Data were entered in MS Excel. Categorical variables were expressed in frequency and percentages. Normality of the continuous variables were tested with Kolmogorov–Smirnov test, and they were found to be not normally distributed. Median and Inter-quartile range was calculated for the continuous variables in each group of patients. Chi-square test and Fishers exact test was applied to test the significance of association between categorical variables. Mann–Whitney test was applied to test the significance in difference in various continuous variables between the 2 groups of patients. A *P* value of < .05 was considered statistically significant.

## Results

Both groups were comparable statistically in terms of demographic profile. The study group was equally distributed between the midazolam and dexmedetomidine groups. The median age of study patients in midazolam group was 8 (IQR 4) and median age in dexmedetomidine group was 8 (IQR 4). Majority of the study participants were males (73.1%). Weight and inter-incisor gap were comparable among the 2-study drug (*P* value .121 and .909, respectively) (Table 2). All patients from both the groups had an ASA of grade 1 and modified Mallampati of grade 2. There was no significant difference in surgical time between the groups (*P* = .248). No significant difference was found between the groups in baseline hemodynamic parameters (*P* > .05). Insertion of PLMA was successful in the first attempt in majority of the patients (96.2%). PLMA was inserted in the second attempt in 5 patients of the midazolam group, though this number was not statistically significant.

**Figure 2.**
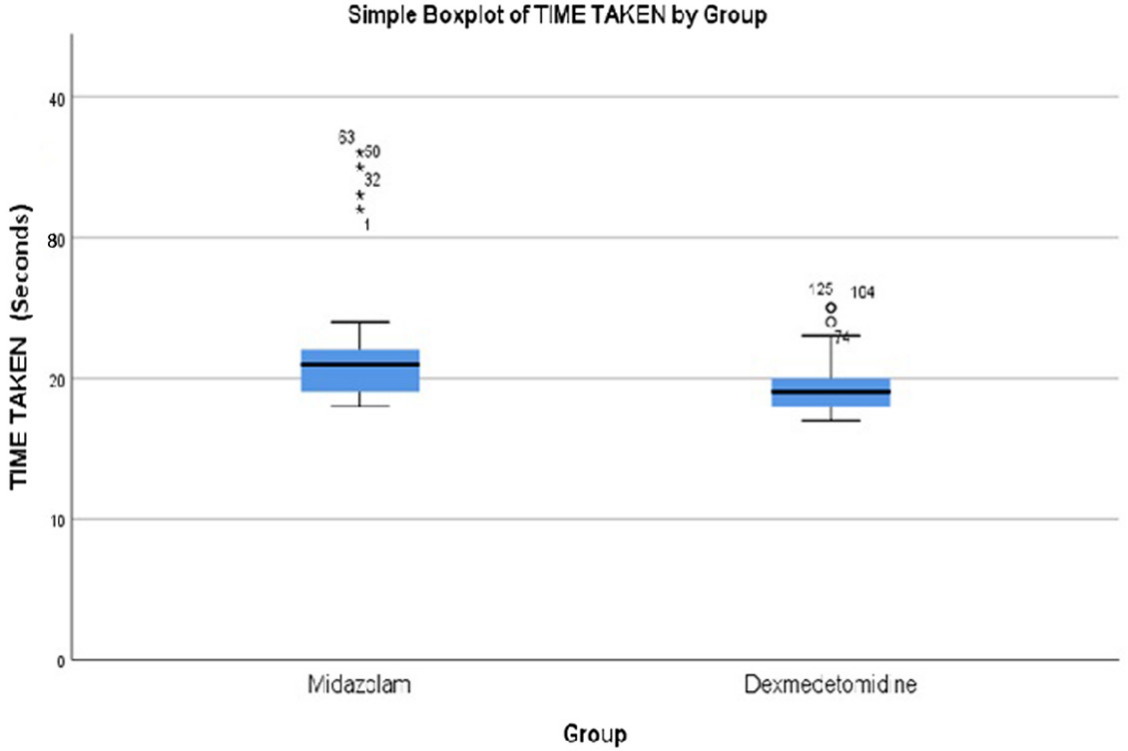
Boxplot of time taken (in seconds) for PLMA insertion by study patients (*P* value .001). * – represents outliners among the midazolam group and ^O^– represents outliners among the dexmedetomidine group.

Time taken was significantly higher among the midazolam group than the dexmedetomidine group (*P* < .001). It was 21 seconds in the midazolam group (interquartile range of 3), whereas it was 19 seconds in the dexmedetomidine group (interquartile range 2) (Figure 2). 

Among the study participants, majority reported excellent Muzi score (53.8%), while 43.1% and 3.1% reported satisfactory and poor scores, respectively. A higher proportion of patients administered dexmedetomidine had excellent Muzi scores in comparison to midazolam (*P* < .001).

Jaw opening, ease of insertion and body movements score were significantly higher in dexmedetomidine as compared to midazolam group (*P* < .05). No significant difference in coughing, gagging or laryngospasm scores was detected among both the study groups (Table 3).

Heart rate was significantly higher among the midazolam group than the dexmedetomidine group at all the time points after study drug (*P* < .001 at all the time points except baseline where it was 0.302). There was no significant difference in mean arterial pressure between the groups at other point of time except at T7, where it was significantly low in dexmedetomidine group than midazolam group (*P* values: baseline = .057, T1 = .184, T2 = .744, T3 = .324, T4 = .664, T5 = .806, T6 = .206, T7 = less than .001).

There was no significant difference in SpO_2_ between the groups at any point of time (*P* = 1.000 at all the time points). Patients in dexmedetomidine group scored significantly higher than the midazolam group in sedation score (*P* < .001). Revised Wong–Baker FACES pain scale scores were significantly higher among the midazolam group than the dexmedetomidine group (*P* < .001).

## Discussion

There have been several studies done on PLMA insertion characteristics with a variety of agents, but fewer studies have been done in paediatric age group. Increased doses of propofol alone cannot control patient’s responses during insertion of LMA. A combination of propofol with other agents will be helpful in tackling this issue.^13^ So in this study, we compared dexmedetomidine with midazolam as a co-induction agent with propofol for insertion characteristics of PLMA.

In our study, the primary objective was to compare the ease of insertion by using first attempt success rate and time taken for PLMA insertion. In our study, the median (interquartile range) time taken for PLMA insertion in dexmedetomidine group was 19 (2) seconds, which is significantly low as compared to midazolam group 21 (3) seconds, although the difference of 2 seconds might not be a clinically significant difference. Gurjar et al^12^ in their study carried out a comparison of midazolam and dexmedetomidine as an adjuvant for PLMA insertion in 100 patients in the age group of 18-60 years of age group. They reported that insertion time for PLMA insertion was significantly less in dexmedetomidine group as compared to midazolam group which is similar to our study result.^12^ Their first time success rate of PLMA insertion was 98% in dexmedetomidine (0.04 µg kg^–1^) and 84% in midazolam group which was significant. In our study, the first attempt success rate in dexmedetomidine group is 100% which could be because we used a higher dose of dexmedetomidine (1 µg kg^–1^) as compared to this study.

The 6-variable scoring system which graded overall insertion characteristics as excellent, satisfactory and poor was also used by Priya et al.^11^ Udaybhaskar et al.^14^ and Sivalingam et al.^15^ In our study, among the 6 parameters of Muzi score of PLMA insertion, jaw opening and ease of insertion was less with more patient movements in midazolam group as compared to dexmedetomidine group, and there were no cases with coughing, gagging and laryngospasm. Hence, in our study, the *P* value of Muzi score is statistically significant, which indicates that dexmedetomidine with propofol provides better condition for PLMA insertion as compared to midazolam. Gurjar and colleagues^13^ had also concluded that resistance to mouth opening, resistance to PLMA insertion, and head and body movements were statistically significant which indicates that dexmedetomidine with propofol produce significant improvement in PLMA insertion characteristics, which is similar to our study results. Farooqy et al^7^ in his comparative study of dexmedetomidine and clonidine as adjuvant to propofol for insertion of laryngeal mask airway concluded that jaw relaxation and overall insertion were better in dexmedetomidine–propofol as compared to clonidine–propofol and coughing, gagging, laryngospasm and involuntary movements were comparable in both groups.

Kavakli et al^16^ had compared the effect of ligocaine and dexmedetomidine before propofol induction during laryngeal mask airway insertion and they concluded that both ligocaine and dexmedetomidine provide better LMA insertion characteristics when used with propofol. Nellore et al in their study on the comparison of dexmedetomidine–propofol versus fentanyl–propofol on insertion condition of PLMA found that dexmedetomidine and fentanyl along with propofol give excellent overall insertion condition with hemodynamic stability, but dexmedetomidine reduces the requirement of induction as well as incremental doses of propofol which is also depicted in our study as there was no case with requirement of incremental propofol dose among dexmedetomidine group.^17^

Our secondary objective was to compare the hemodynamics, recovery characteristics and post-operative pain assessment. In our study, baseline heart rate (HR) was comparable in both the groups. We observed that there was statistically significant fall in HR among the 2 groups after giving the study drug. However, fall in HR was more in dexmedetomidine group as compared to midazolam. Post–induction, mean arterial blood pressure was comparatively low at all the time points in dexmedetomidine group as compared to midazolam group, but this difference was statistically not significant, except at T7, where the mean arterial blood pressure was significantly low in dexmedetomidine group as compared to midazolam group. This could be due to peak effect of the drug at T7. Mechanism for reduced HR during dexmedetomidine infusion may be due to an increase in vagal tone and reduction in sympathetic drive, but there was decrease in mean arterial blood pressure by inhibition of noradrenaline and central sympathetic activity. In our study, post-operative sedation score (Ramsay sedation score) and revised Wong-Baker FACES pain scale were significantly more in midazolam group as compared to dexmedetomidine group despite using same analgesic in both the groups. Smooth emergence was also noted in the dexmedetomidine group as compared to midazolam group. Choudhary et al^18^ in their study on dexmedetomidine with propofol versus fentanyl with propofol for insertion of Proseal LMA also found similar post-operative sedation properties.

### Strengths

As selection and classification of patients into the 2 groups was randomised, it decreased selection bias and minimised confounding. As the present study was conducted in a controlled environment, measurements (like hemodynamics, duration of analgesia and sedation could be captured precisely and consistently at different time points and accuracy of parametric data was ensured. As patients were not aware about their group allocation, the patient or observer bias was low or minimal. The response rate was more than 90%.

### Limitations

As sample size was small (n = 130), the differences or effect size observed could have been higher or lower in actual clinical practice. We had limited ability to detect any rare side effects or adverse events of the tested anaesthetic treatment due to the low sample size.

## Conclusion

The study population was comparable demographically in terms of age, sex, weight, ASA grading, modified Mallampatti grading and inter-incisor gap. First attempt success rate was high in dexmedetomidine and time of insertion was less as compared to midazolam group. Dexmedetomidine (at dose 1 µg kg^–1^) as compared to midazolam (20 µg kg^–1^) produces better insertion characteristics for PLMA when used as co-induction agent with propofol in terms of jaw opening, ease of insertion, coughing, gagging, patient movement and laryngospasm. Dexmedetomidine provides better haemodynamic stability intra-operatively and better sedation and smooth emergence post-operatively as compared to midazolam. We conclude that dexmedetomidine is better than midazolam when used as a co-induction agent with propofol for insertion characteristics of PLMA and hemodynamic stability.

## Figures and Tables

**Table 1.** Modified Muzi Score for Grading of Conditions for PLMA Insertion

**SCORE**	**3**	**2**	**1**
Introduction of PLMA			
Jaw opening	Full	Partial	Nil
Ease of insertion	Easy	Difficult	Impossible
Patient response			
Coughing	Nil	Minor	Severe
Gagging	Nil	Minor	Severe
Patient movement	Nil	Moderate	Vigorous
Laryngospasm	Nil	Partial	Total

Total score: 18 = excellent, 16-17 = satisfactory, less than 16 = poor.

PLMA, ProSeal laryngeal mask airway.

**Table 2.** Demographic Data

Patient factors	Group 1 (65)	Group 2 (65)	*P*
Median age in years (IQR)	8 (4)	8 (4)	.91^α^
Gender (male/female)	45/20	50/15	.32^β^
Median weight in kg (IQR)	21 (10)	26 (9.5)	.12^α^
Median surgical time in minutes (IQR)	45 (10)	60 (30)	.24^α^
Inter-incisor gap in centimetres (IQR)	4 (0.55)	4 (0)	.90^α^

Group 1 = midazolam Group, group 2 = dexmedetomidine group, α and β represent test of significance used to calculate P value (α = Mann–Whitney, β = chi-square test).

IQR, interquartile range.

**Table 3.** Muzi Score Parameters

**Parameters**	**Group**	**Sample Size**	**Median Score**	**Interquartile Range**	***P***
Jaw opening	Midazolam	65	3	3,3	<.001
	Dexmedetomidine	65	0	0,0	
Ease of insertion	Midazolam	65	3	3,3	.043
	Dexmedetomidine	65	3	3,3	
Coughing	Midazolam	65	3	3,3	1.000
	Dexmedetomidine	65	3	3,3	
Gagging	Midazolam	65	3	3,3	1.000
	Dexmedetomidine	65	3	3,3	
Patient movement	Midazolam	65	3	3,3	<.001
	Dexmedetomidine	65	3	3,3	
Laryngospasm	Midazolam	65	3	3,3	1.000
	Dexmedetomidine	65	3	3,3	
